# Clinical profile and comorbidity of traumatic brain injury among younger and older men and women: a brief research notes

**DOI:** 10.1186/s13104-017-2682-x

**Published:** 2017-08-08

**Authors:** Vincy Chan, Tatyana Mollayeva, Kenneth J. Ottenbacher, Angela Colantonio

**Affiliations:** 1 Toronto Rehabilitation Institute-University Health Network, Toronto, Ontario Canada; 2 Rehabilitation Sciences Institute, Faculty of Medicine, University of Toronto, Toronto, Ontario Canada; 3 Division of Rehabilitation Science, Center for Recovery, Physical Activity and Nutrition, School of Health Professions, University of Texas Medical Branch, Galveston, Texas USA

**Keywords:** Traumatic brain injury, Inpatient rehabilitation, Sex, Age, Index disease, Comorbidity, Multimorbidity, Prevalence

## Abstract

**Objective:**

Comorbid disorders influence the course and outcomes of rehabilitation following traumatic brain injury (TBI), yet sex- and age-related disparities in the frequency distribution of these disorders remain poorly understood. We aimed to describe comorbid disorders by the International Classification of Diseases in patients with TBI undergoing inpatient rehabilitation in Ontario, Canada over a 3-year period, by sex and age, and discuss their potential impact on rehabilitation outcomes.

**Results:**

The percentage of TBI patients with one or more comorbid disorder is higher among older (≥65 years) men and women than among those who are younger or middle-aged (<65 years). Among younger and middle-aged patients, multiple injuries and trauma, mental health conditions, and nervous system disorders were the most prevalent comorbidities. In older patients, circulatory, endocrine, nutritional, metabolic, and immune disorders were the most prevalent comorbidities. Our results suggest that a multisystem view of rehabilitation of men and women with TBI across age categories is needed to reflect the complex clinical profile of TBI patients undergoing rehabilitation.

**Electronic supplementary material:**

The online version of this article (doi:10.1186/s13104-017-2682-x) contains supplementary material, which is available to authorized users.

## Introduction

Traumatic brain injury (TBI), defined as “a traumatically induced structural injury and/or physiological disruption of brain function as a result of an external force” [1], remains a significant public health issue [2, 3]. Recent initiatives in TBI care highlight the complexity of its clinical management, and recommend that an assessment of co-existing (comorbid) disorders be included [4]. “Comorbidity” refers to any disease co-existing with an index disease [5]; it can alter the clinical course of patients by affecting selections of healthcare services and outcomes [6, 7]. While previous population-based research from United States and Canada [8–12] has documented comorbidities in TBI, there has been no comprehensive description of sex- and age- specific clinical nosologies for TBI patients entering inpatient rehabilitation. Historically, TBI has been regarded as an injury mostly affecting young males [13]. However, recent epidemiological trends highlight older females being as frequently affected as males, largely as a result of falls [13]. Because many diseases affecting males and females have different frequencies and presentations across their lifespan [14, 15], understanding the variability of comorbidity in rehabilitation [12, 14, 15] will likely lead to a still greater recognition of the unique sex-specific facets of TBI [16, 17]. Therefore, the aims of this study were to: (1) describe comorbidity frequencies (along the spectrum of disorders) in younger and older patients, by sex; (2) assess possible sex differences in comorbid disorder frequencies; and (3) offer an evidence-based discussion of the clinical relevance of the most frequent comorbidity in TBI.

## Main text

### Methods

#### Participants

This population-based retrospective cohort study included all patients admitted to inpatient rehabilitation facilities in Ontario, Canada between the fiscal years 2004/05 and 2007/08. Data from all acute care inpatient rehabilitation units and freestanding rehabilitation hospitals were obtained from the National Rehabilitation Reporting System (NRS) [18]. To assess sociodemographic variables, we analyzed primary language (English versus other) and sex (male versus female). The FIM™ Instrument assessed patients’ physical and cognitive ability, with total scores ranging from 18 to 126, motor scores ranging from 13 to 91 whereas the cognitive scores ranging from 5 to 35, where lower scores represent ‘total dependence’ and higher scores ‘total independence’ [19]. The length of stay (LOS) represents the number of days between admission and discharge, serving as a proxy for injury severity and comorbidity load [18]. Assessed comorbid categories and subcategories are listed in Additional file 1: Appendix S1.

#### Statistical analysis

We described continuous data with means and standard deviation, or medians and ranges, and categorical data with frequency counts. Depending on the type of data, we used t tests for binary continuous variables, Chi square tests when analyzing differences between comorbidity categories in both sexes, and the Fisher’s exact test when the expected value of any cell of a contingency table was below five. Separate analyses were conducted for younger (<65 years old) and older (≥65 years old) adults. To address the likelihood of multiple comparison fallacy, we used the Bonferroni method. Two-sided p values of <0.05 were considered statistically significant. All analyses were performed using SAS v. 9.1 (SAS Institute, Cary, NC).

### Results

#### Patient profile

Table 1 outlines the study population’s demographic and clinical data (n = 1791). Of all patients undergoing TBI inpatient rehabilitation, 70.2% were males. The older patient group’s sex distribution was 57.3% male and 42.7% female. The mean LOS/median LOS was 49 (SD 50.8)/37 days, and 45 (SD 40.8)/36 days for male and female patients, respectively. LOS differences between sexes in all age categories were non-significant. Male patients had higher total functional scores and motor rating than females at admission and at discharge. When stratified by age, sex differences remained significant in the total functional score at admission (p < 0.001) and discharge (p = 0.013), motor score at admission (p < 0.001) and discharge (p = 0.002), and FIM efficiency (p = 0.048), in younger patients only.

**Table 1:** Descriptive characteristics of TBI patients in inpatient rehabilitation

Characteristics	Total (N = 1791)			0–64 years (N = 1164)			65+ years (N = 627)		
	Males N = 1257 (70.2%)	Females N = 534 (29.8%)	p value	Males N = 898 (77.1%)	Females N = 266 (22.9%)	p value	Males N = 359 (57.3%)	Females N = 268 (42.7%)	p value
Demographic variables									
Age (years)	50.1 ± 20.9	59.8 ± 21.8	<0.001	39.5 ± 14.3	41.2 ± 14.4	0.089	76.5 ± 6.7	78.3 ± 7.2	0.001
English language^a^	1148 (91.3)	486 (91.0)	0.828	840 (93.5)	250 (94.0)	0.794	308 (85.8)	236 (88.1)	0.408
Clinical variables									
1+ comorbid condition; admission^a^	1038 (82.6)	482 (90.3)	<0.001	716 (79.7)	226 (85.0)	0.057	322 (89.7)	256 (95.5)	<0.001
1+ comorbid condition; discharge^a^	168 (13.4)	96 (18.0)	0.037	105 (11.7)	33 (12.4)	0.752	63 (17.6)	63 (23.5)	0.066
Length of stay (days)	48.8 ± 50.8 Q_2_ = 37	45.4 ± 40.8 Q_2_ = 36	0.134	54.0 ± 56.5 Q_2_ = 41.5	56.4 ± 48.5 Q_2_ = 45	0.490	35.9 ± 28.7 Q_2_ = 31	34.5 ± 27.5 Q_2_ = 28	0.530
Total function score, FIM™ instrument; admit	83.1 ± 28.1 Q_2_ = 90	78.4 ± 25.9 Q_2_ = 83	<0.001	84.9 ± 29.5 Q_2_ = 94	79.3 ± 29.3 Q_2_ = 87	0.007	78.6 ± 23.5 Q_2_ = 83	77.4 ± 22.1 Q_2_ = 80	0.510
Total function score, FIM™ instrument; discharge	104.2 ± 24.2 Q_2_ = 114	101.1 ± 24.7 Q_2_ = 110	0.013	105.9 ± 24.5 Q_2_ = 116	104.1 ± 25.8 Q_2_ = 114	0.294	99.9 ± 22.8 Q_2_ = 108	98.0 ± 23.0 Q_2_ = 106	0.314
Total motor rating, FIM™ instrument; admit	60.3 ± 23.8 Q_2_ = 65	54.9 ± 21.6 Q_2_ = 56	<0.001	62.8 ± 24.8 Q_2_ = 70	57.2 ± 24.4 Q_2_ = 62	0.001	54.1 ± 19.6 Q_2_ = 56	52.6 ± 18.2 Q_2_ = 53	0.338
Total motor rating, FIM™ instrument; discharge	77.2 ± 19.5 Q_2_ = 86	74.1 ± 19.6 Q_2_ = 81	0.002	78.9 ± 19.7 Q_2_ = 88	76.8 ± 20.6 Q_2_ = 85	0.138	72.9 ± 18.2 Q_2_ = 80	71.2 ± 18.1 Q_2_ = 78	0.251
Total cognitive Rating, FIM™ instrument; admit	22.8 ± 7.8 Q_2_ = 24	23.5 ± 7.7 Q_2_ = 24	0.103	22.1 ± 7.8 Q_2_ = 23	22.1 ± 8.0 Q_2_ = 23	0.999	24.6 ± 7.5 Q_2_ = 25	24.8 ± 7.2 Q_2_ = 25	0.670
Total cognitive rating, FIM™ instrument; discharge	27.2 ± 6.7 Q_2_ = 29	27.2 ± 6.8 Q_2_ = 29	0.939	27.2 ± 6.7 Q_2_ = 29	27.3 ± 6.8 Q_2_ = 29	0.839	27.1 ± 6.8 Q_2_ = 28	27.1 ± 6.8 Q_2_ = 29	0.967
FIM efficiency	0.6 ± 1.0	0.7 ± 0.9	0.052	0.5 ± 0.9	0.6 ± 0.6	0.048	0.9 ± 1.0	0.8 ± 1.1	0.370

Patients with traumatic brain injury in inpatient rehabilitation between 2004/05 and 2007/08 in Ontario, Canada: characteristics by sex and age groups

Values are mean ± SD or N (%) unless otherwise specified. T tests, except ^a^ Chi squared tests. Q_2_ indicates the median value

#### Comorbidities

**Fig. 1:**
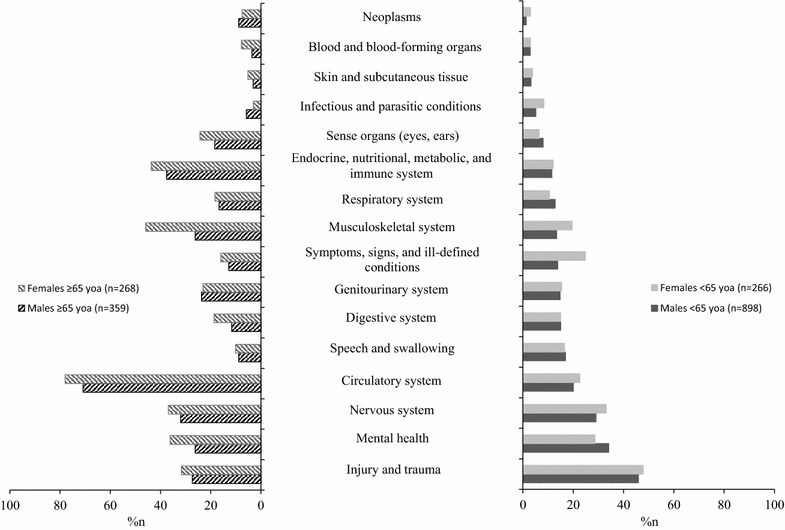
ICD-10 categories of comorbid conditions in younger and older males and females with TBI. Categories of comorbid conditions in younger [<65 years of age (yoa)] and older (≥65 yoa) males and females with TBI in inpatient rehabilitation in Ontario, Canada 2004/05–2007/08. For each patient, multiple comorbidities were included. Regardless of age, sex differences at the p < 0.001 after Bonferroni correction were observed in disorders of the Endocrine, Nutritional, Metabolic and Immune System, Circulatory System, Musculo-skeletal system, and the Symptoms, Signs, and Ill-defined conditions. In the younger age group, sex differences at the p < 0.001 after Bonferroni correction were observed in Symptoms, Signs, and Ill-defined conditions, and in the older age group, in disorders of the Musculo-skeletal system

Figure 1 presents comorbid conditions by age and sex. A significantly higher proportion of female patients had at least one comorbid disorder at admission and discharge (p < 0.001) compared to males. When stratified by age, results remained significant only at admission and only in older females (p < 0.001).

The most common comorbidities among *male patients*, regardless of age, were disorders of multiple injury and trauma (40.6%), the circulatory system (34.5%), mental health (31.8%), and the nervous system (29.8%). Among *female patients*, regardless of age, the most common comorbidities related to the circulatory system (50.4%), multiple injury and trauma (39.7%), the nervous system (35.0%), and the musculoskeletal system (32.8%).

A significantly higher percentage of females than males experienced conditions of the endocrine, nutrition, metabolism, and immune systems (p < 0.001), the circulatory system (p < 0.001), and the musculoskeletal system (p < 0.001).

#### Results stratified by age

The most common comorbidities in *younger* patients of both sexes included multiple injury and trauma (45.9% male; 47.8% female); mental health (34.1%, male; 28.6% female); and the nervous system (29.0% male; 33.1% female). Younger female patients more frequently experienced comorbidities related to symptoms, signs, and ill-defined conditions (i.e., chapter 18 of the ICD-10, which includes symptoms, signs, abnormal results of clinical/investigative procedures, and ill-defined conditions regarding which no diagnosis is recorded) compared to males (p < 0.001).

In *older* patients, circulatory system comorbidities were the most common in both sexes. In older men, comorbidities of the endocrine, nutrition, metabolism, and immune systems (37.6%); the nervous system (32.0%); and injury and trauma (27.3%) were common. Older female patients experienced more frequent comorbid conditions of the musculoskeletal system (45.9%); and endocrine, nutrition, metabolism, and immune systems (43.7%). A significantly higher proportion of older females had musculoskeletal conditions compared to older males (p < 0.001).

### Discussion

Most previous studies on TBI comorbidity that employed pre-defined checklist or ICD classification codes of comorbid disorders identified increased comorbidity frequencies in TBI patient populations across continuum of care and age [8–12]. Our study’s methodological novelty lies in the comparison of population-derived data for each comorbid disorder in TBI by sex within younger and older age categories. Although our results generally confirmed those of earlier reports (high prevalence of cardiovascular pathology [9], psychiatric/behavioural disorders [8, 12], musculoskeletal disorders [8], substance use disorders [10–12], etc.), we generated a qualitatively enriched comorbidity profile that includes (1) details on the magnitude of comorbidity prevalence in patients with TBI undergoing rehabilitation, (2) sex differences in the comorbidity across age categories, and (3) specificity of age-dependent comorbidity in TBI. By positioning our results in light of previous research on age- and sex specific comorbidity, we were able to highlight comorbid disorders of high relevance to TBI patients undergoing rehabilitation, and shed light on how age and sex may account for the observed results.

*Musculoskeletal system comorbidities* were observed more frequently in female patients than in male patients, regardless of age. This is unsurprising: osteoarthritis, one of the most commonly occurring disorders within this category, is more prevalent in females [20]. An earlier study reported that 37% of community-dwelling older adults have arthritis, and the causality of this comorbid condition and TBIs, especially in older women resulted from falls, should be considered [21, 22].

The high frequency of *nervous system disorders* we observed is consistent with previous studies [5, 8–11]. Chen and colleagues reported an increased stroke risk in individuals with TBI, with a 10-fold higher risk at 3 months post-injury [23]. Because stroke is a highly disabling condition, this relationship demonstrates the need for significant attention and care. Similarly, in a population-based study by Macciocchi and colleagues, 60% of patients with a traumatic spinal cord injury had a co-occurring TBI [24]. Paraplegic patients with severe TBI had lower FIM™ motor scores and a longer LOS in rehabilitation, compared to those without TBI or mild TBI [25]. Additional injury to the peripheral nerves occurs in approximately 34% of patients with severe TBI [26]. As the symptoms of neuropathy are similar to those of the central nervous system disorders, diagnoses and interventions should be assigned with care.

Like reported in earlier studies, *mental health* disorders are prevalent. In individuals with mild or moderate/severe TBI, an increased prevalence of 34 and 49% respectively, for any psychiatric condition has been reported during the first year post-injury [27]. Affective disorders are associated with poor functional outcomes [28]. An increased risk of psychosis has been reported in patients with moderate to severe head injuries, with age at injury having no effect on the magnitude of risk [29]. Arciniegas and colleagues reported that male patients are at a greater risk of developing psychosis following TBI, compared to females [30]. Psychosis is a potential confounder of TBI diagnosis, and is therefore relevant to rehabilitation.

*Circulatory system disorders* after brain injury, specifically neurogenic cardiovascular abnormalities, have been linked to increased morbidity and mortality [31]. TBI is reported to trigger biochemical events by altering cerebral blood flow, which has been associated with poorer neurological outcomes [32]. Researchers reported that even mild TBI is associated with the presence and severity of coronary atherosclerosis and predicts cardiovascular mortality independent of age, sex and other risk factors [33]. Therefore, a comprehensive stratification of risk and circulatory system disorder management in TBI patients undergoing inpatient rehabilitation are needed.

*Metabolic* disorders are more prevalent in older patients compared to their younger counterparts. In a study of patients with moderate to severe TBI, those with diabetes mellitus had a significantly longer hospital stay and higher mortality rate than those without [34]. Clinical malnutrition, a notable comorbidity, has been associated with outcomes following severe TBI 6 months post-injury, independent of the Glasgow Coma Scale score [35]. *Neuroendocrine* dysfunction is prevalent in approximately two-thirds of people with TBI. Having been shown to result in a complex cascade of pathophysiological and neurochemical events, TBI concerns adversities towards both recovery and stress level, and therefore warrants attention [36, 37]. Disruption of neural connections between the cerebral cortex and the brainstem due to TBI frequently impairs bladder control [38]. This is consistent with the high rate *of genitourinary comorbidities* in older males and females in our study. Common *ill*-*defined symptoms* following TBI involve sleep, pain, apathy, and fatigue, often at higher rates than those observed in the general population [39]. These symptoms can impede rehabilitation by reducing effectiveness and compliance with treatment, and increasing the LOS.

Our study has a number of strengths. Our effort encompassed inclusive adult inpatient population with TBI over a 3-year period. This is the greatest advance over earlier research on the topic of comorbidity, as the NRS includes all data from rehabilitation hospitals from a publicly funded system of care where reporting inpatient rehabilitation data is mandatory. In addition, the use of standardized coding makes our findings more accurate than those based on self-reported data.

In conclusion, our study illustrates that patients have complex comorbidities during rehabilitation, a reality which must be addressed. To develop patient-oriented rehabilitation programs and support clinical decisions, further characterization of comorbidity in TBI in relation to patient-related and system-related outcomes is warranted. In addition, more advanced statistical methodologies that allow modeling of complex interactions of comorbid disorder clusters, starting at the time of injury event, has enormous potential for advancing our understanding of one of the most complex injury, TBI. Finally, because female patients represented a smaller proportion of rehabilitation service users in both younger and older age categories, but more frequently exhibited one or more comorbid disorders in inpatient rehabilitation compared to that of male patients, it is crucial to consider sex inequalities and vulnerabilities when designating resources.

## Limitations

- Information such as acute management of TBI was not available in the NRS; significant limitation because acute care narrates to characteristics of patients entering rehabilitation.
- We utilized the earliest NRS dataset using ICD-10 codes to capture TBI and comorbidity; a future study is planned to determine whether the past decade has seen a change in the frequency distribution of TBI and comorbidity in males and females across age categories.
- We utilized age 65 for defining “old” because of many legislative and healthcare services use this cut-off score; future study should utilize smaller age categories to determine which comorbid disorders are important in the test of age-related hypotheses.

## Additional file

**Additional file 1: Appendix S1.** Comorbidity categories and corresponding conditions. Comorbidity categories and corresponding conditions by ICD-10 chapter headings.
