# Supplementary material for: Clinical profile and comorbidity of traumatic brain injury among younger and older men and women: a brief research notes
Source: BMC Res Notes. 2017 Aug 8;10:371. doi: 10.1186/s13104-017-2682-x (PMC5549298; doi:10.1186/s13104-017-2682-x)
Supplement: Supplementary file 1 — Additional file 1: Appendix S1. Comorbidity categories and corresponding conditions. Comorbidity categories and corresponding conditions by ICD-10 chapter headings. [file 13104_2017_2682_MOESM1_ESM.docx]

| ***Comorbidity Category*** | **Conditions** |
| --- | --- |
| *Blood and blood-forming organs* | Anemia |
|  | Coagulation defects |
|  | Disorders of blood forming organs, excludes leukemia |
| *Circulatory system* | Cardiac conditions |
|  | Vascular conditions |
| *Digestive system* | Colostomy |
|  | Gastroenteritis (includes diarrhea) |
| *Endocrine, nutritional, metabolic, and immune system* | Diabetes |
|  | Malnutrition |
|  | Thyroid disorders |
|  | Obesity |
| *Genitourinary system* | Urinary tract infection |
|  | Renal failure |
| *Infectious and parasitic conditions* | Primary tuberculosis infection |
|  | Viral hepatitis |
|  | HIV with AIDS |
|  | Acute poliomyelitis |
| *Injury and trauma* | Intracranial injury |
|  | Spinal cord injury |
|  | Nerve injury |
|  | Fractures and dislocations |
|  | Joint sprains and strains |
|  | Burns |
|  | Traumatic amputation |
|  | Open wounds of limbs |
|  | Internal injury |
|  | Superficial injury and contusions |
|  | Multiple trauma and crushing injury |
| *Mental health* | Dementia |
|  | Affective disorders |
|  | Alcohol/drug dependence |
|  | Neurotic disorders |
|  | Paranoid states |
|  | Schizophrenic psychoses |
| *Musculoskeletal system* | Arthritic/connective tissue disorders |
|  | Joint/soft tissue disorders (non-injury) |
|  | Joint replacement |
|  | Amputation |
|  | Pain |
| *Neoplasms* | Malignant/benign neoplasms |
|  | Tumor/cancers |
| *Nervous system* | Stroke |
|  | Spinal cord |
|  | Viral encephalitis |
|  | Alzheimer’s disease |
|  | Parkinson’s diseases |
|  | Bacterial meningitis |
|  | Cranial/peripheral nerve injury |
| *Respiratory system* | Viral/bacterial pneumonia |
|  | Bronchitis |
|  | Asthma |
|  | Cystic fibrosis |
|  | Respiratory failure, includes acute and chronic |
| *Sense organs (eyes, ears)* | Visual loss |
|  | Hearing loss |
| *Skin and subcutaneous tissue* | Psoriasis and similar disorders |
|  | Chronic ulcer of the skin |
|  | Scar condition including fibrosis of skin |
|  | Cellulitis and abscess |
| *Speech and swallowing* | Aphasia (includes impaired speech) |
|  | Other speech disorders (includes dysphasia, swallowing disorders, dysarthria) |
|  | Voice disturbance (includes hoarseness) |
| *Symptoms, signs, and ill-defined conditions* | Central nervous system complications |
|  | General symptoms – alteration of consciousness, including coma, vegetative state, stupor |
|  | Abnormality of gait, involuntary movements and/or lack of coordination |
|  | Disturbances of skin sensation, cyanosis and pallor |
|  | Headaches, lightheadedness, dizziness, vertigo |
|  | Heartburn, nausea, vomiting |
|  | Weight loss/gain, excluding aphasia |
